# A hotspot for new genes

**DOI:** 10.7554/eLife.50136

**Published:** 2019-08-29

**Authors:** Anne-Marie Dion-Côté

**Affiliations:** Département de biologieUniversité de MonctonMonctonCanada

**Keywords:** de novo gene, mutational load, single-cell sequencing, expression dynamics, *Drosophila*, spermatogenesis, *D. melanogaster*

## Abstract

Single-cell RNA sequencing in fruit flies gives an unprecedented picture of how new genes are expressed during the formation of sperm.

**Related research article** Witt E, Benjamin S, Svetec N, Zhao L. 2019. Testis single-cell RNA-seq reveals the dynamics of de novo gene transcription and germline mutational bias in *Drosophila*. *eLife* **8**:e47138. doi: 10.7554/eLife.47138

New genes can be produced in a number of different ways. Existing genes can be duplicated, while frameshift mutations may change the way a cell reads a sequence, which could lead to new proteins. 'De novo' genes can also be created from previously non-coding sequences. It was thought until recently that the emergence of these genes was extremely rare, but the advent of modern genomics and transcriptomics has revealed that this is not the case (Schlötterer, 2015; Jacob, 1977). In multicellular organisms, only a small proportion of the genome codes for proteins, yet a large fraction of the rest of the genome is actively transcribed and translated (Clark et al., 2011; Ruiz-Orera et al., 2018). These non-coding sequences provide the raw material for natural selection to act upon and create new genes: considering that most of the genome is non-coding in multicellular organisms, the potential for de novo genes to emerge is enormous.

De novo genes tend to be expressed in the testis and involved in male reproductive processes (Levine et al., 2006; Begun et al., 2007; Zhao et al., 2014). However, many of these genes are not fixed within a species, which means they are susceptible to disappear. In addition, a large number of genes associated with male reproduction are rapidly evolving (Swanson et al., 2001). These observations are surprising given that sperm formation is a complex, highly conserved process. It is possible that de novo genes arise more often in the testis because future sperm cells have a permissive DNA state which would allow transcription to proceed less specifically than in other tissues, exposing non-coding sequences to selection (Kaessmann, 2010). However, this hypothesis is notoriously difficult to test because the testis is a highly heterogeneous tissue: for example, germ cells pass through many different stages before they mature into sperm.

Single-cell RNA sequencing is a powerful technology that circumvents the challenges associated with tissue heterogeneity by revealing the expression profile of individual cells. It can be harnessed to study mixed cell populations in tumors, or to track transcriptional dynamics during complex developmental processes such as sperm formation. This mechanism, also known as spermatogenesis, requires a pool of germ stem cells to undergo a series of mitotic and meiotic divisions to finally produce mature sperm (Figure 1). Now, in eLife, Li Zhao and colleagues at the Rockefeller University – including Evan Witt as first author, Sigi Benjamin and Nicolas Svetec – report having leveraged single-cell RNA sequencing to investigate how de novo genes are expressed in fly testis during the different stages of spermatogenesis (Witt et al., 2019).

**Figure 1.**
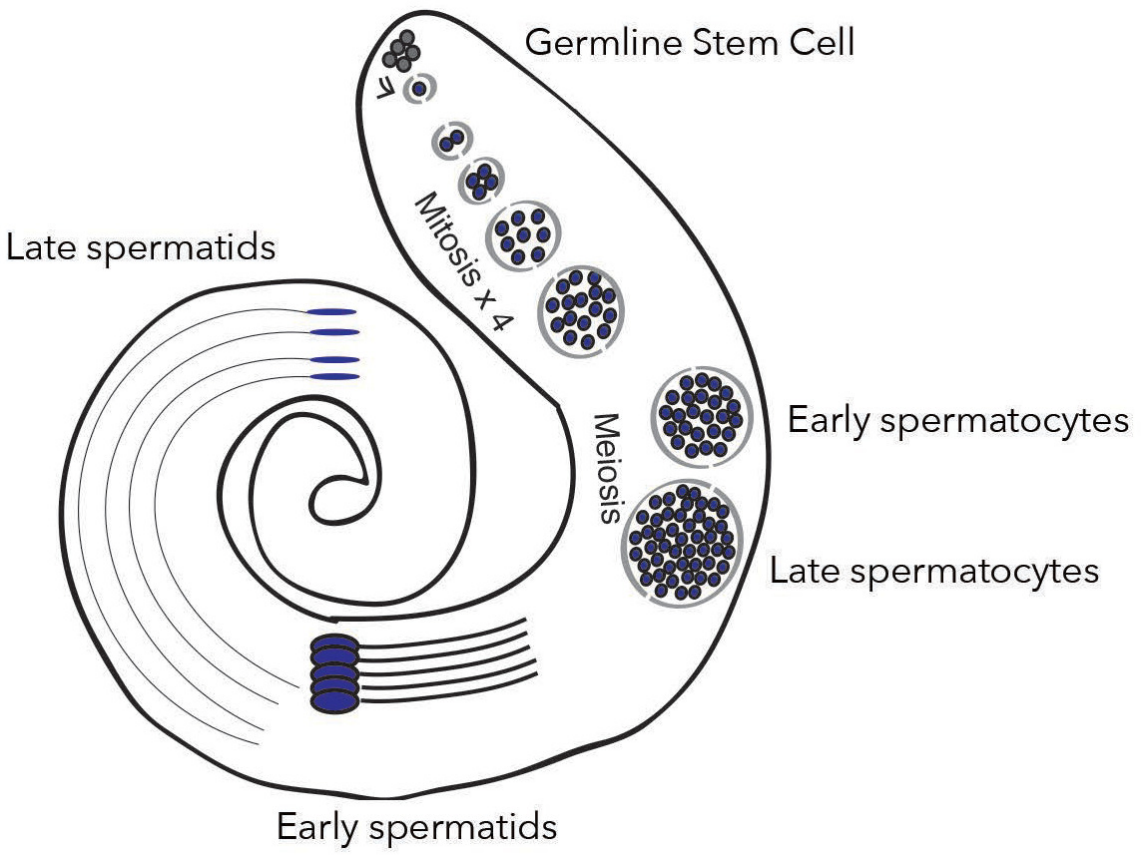
New genes are expressed differently depending on spermatogenesis stages. In the testis of fruit flies, the creation of mature sperm, or spermatogenesis, starts with a germline stem cell going through several rounds of mitosis to form early spermatocytes. After meiosis, these cells become late spermatocytes, which then develop into early and late spermatids. Witt et al. show that fixed de novo genes, which emerge from non-coding sequences, are expressed during mid-spermatogenesis, in particular in early spermatocytes. In contrast, other types of new genes, for example which come from gene duplication, are expressed at different stages.

Image credit: Witt et al., 2019; adapted from Figure 1A (CC BY 4.0).

First, the Rockefeller team was able to categorize the cells as germline stem cells, somatic cells, or cells going through specific stages of maturation by analyzing the expression of well-known spermatogenesis marker genes. In addition, the researchers used overall transcriptional changes to reconstruct an inferred ‘pseudotime’, a roadmap of the different stages that cells go through as they develop into sperm. Together, these approaches allowed Witt et al. to thoroughly document the gene expression profiles of specific cell types during spermatogenesis.

In particular, they found that fixed de novo genes (that is, de novo genes that are unlikely to disappear from a species) were expressed differently depending on developmental stage. For example, they were more often switched on in meiotic germ cells, a pattern reminiscent of canonical genes expressed only in testes. However, unfixed de novo genes were less expressed in germline stem cells, and enriched in early sperm cells (which have gone through meiosis). These results indicate that de novo genes could be expressed during meiosis, and that the expression pattern of a de novo gene may impact its probability to reach fixation.

Next, Witt et al. compared the expression profiles of de novo and recent duplicate genes. The expression pattern of almost half of the derived duplicate genes was biased towards early and late spermatogenesis, a pattern never observed for parental genes. On the contrary, the expression of the majority of de novo genes was biased towards mid-spermatogenesis. These observations suggest that de novo and young duplicate genes are regulated differently, and that the mode of emergence of a new gene constrains how it is expressed.

Finally, Witt et al. developed a method to infer the presence of mutations from single-cell RNA sequencing data. Combined with the cell type and pseudotime information, this allowed them to estimate the mutational load – the amount of potentially deleterious mutations – over spermatogenesis. They found that the mutational load tends to decrease over pseudotime, suggesting that DNA damage was repaired or that carrier cells were eliminated. While this finding requires further validation, it opens the door to many questions related to DNA repair dynamics, selection pressure within the testis and even male fertility.

As evolution largely relies on the mutation rate in the germline, the work by Witt et al. elegantly highlights how single-cell sequencing technologies can start to address long-standing questions in evolutionary biology. One of the upcoming challenges is now to integrate these highly multidimensional data with the complex molecular pathways involved in reproduction and development.
